# Genetic Variability and Aggressiveness of *Tilletia indica* Isolates Causing Karnal Bunt in Wheat

**DOI:** 10.3390/jof8030219

**Published:** 2022-02-23

**Authors:** Aasma, Shahzad Asad, Muhammad Fayyaz, Khawar Majeed, Aziz ur Rehman, Sajid Ali, Jindong Liu, Awais Rasheed, Yamei Wang

**Affiliations:** 1 Department of Plant and Environmental Protection, Crop Disease Research Institute, National Agriculture Research Center, Islamabad 44000, Pakistan; aasmafahad@outlook.com (A.); asadtaimoor@yahoo.com (S.A.); sofayyaz@yahoo.com (M.F.); 2 Department of Plant Sciences, Quaid-i-Azam University, Islamabad 45320, Pakistan; khawar860@gmail.com; 3 Wheat Research Institute, Ayub Agriculture Research Institute, Faisalabad 38850, Pakistan; aziz_kml@yahoo.com; 4 Department of Agriculture, Hazara University, Mansehra 21120, Pakistan; bioscientist122@yahoo.com; 5 Institute of Crop Science, Chinese Academy of Agricultural Sciences (CAAS), 12 Zhongguancun South Street, Beijing 100081, China; liujindong@caas.cn; 6 Shenzhen Branch, Guangdong Laboratory for Lingnan Modern Agriculture, Genome Analysis Laboratory of the Ministry of Agriculture and Rural Affairs, Agricultural Genomics Institute at Shenzhen, Chinese Academy of Agricultural Sciences, Shenzhen 518120, China; 7 School of Agriculture, Sun Yat-sen University, Shenzhen 510275, China

**Keywords:** Karnal bunt, *Tilletia indica*, virulence, genetic variability, bread wheat, fungal disease

## Abstract

Karnal bunt caused by *Tilletia indica* is a quarantine disease of wheat causing huge economic losses due to the ban on the import of bunted grains. This study was designed to characterize pathogenicity, aggressiveness and genetic diversity of 68 *Tilletia indica* isolates collected from different geographic regions of Pakistan. Forty-six isolates were tested for their pathogenicity on eight wheat varieties, out of which three were non-aggressive. The coefficient of infection (CI) ranged from 15.73% (PB-25) to 10% (PB-68, PB-60, and PB-43). The isolates collected from central Punjab showed higher infestation compared to other isolates. Among the wheat varieties used for the aggressiveness study, WL-711 showed susceptible reaction with 10.88% CI, while NIFA-Barsat, HD-29, Janbaz, Bakhtawar-92, Tatara, and AARI 2011 showed resistance to the highly resistant response. These isolates were amplified using 31 random amplified polymorphic DNA (RAPD) markers and 32 inter-simple sequence repeat (ISSR) markers for diversity analysis. The principal component analysis (PCA) and analysis of molecular variance (AMOVA) showed greater divergence among isolates collected from Punjab and Khyber Pakhtunkhwa (KPK), with a moderate level of admixture. The isolates from Faisalabad (Punjab) were more aggressive compared to isolates from KPK and were clearly separated based on PCA, indicating the significant genetic distance in the populations. Our findings will assist breeders and pathologists in better understanding the pathogenic variability in *Tilletia indica* and in subsequent disease management.

## 1. Introduction

The pathogen, *Tilletia indica (T. indica)*, causes Karnal bunt (KB) in wheat, durum wheat, and triticale, and is also named as a partial bunt. It is an internationally quarantined disease [1] affecting quality and production of wheat, and there is zero tolerance in various countries against KB for importing bunted wheat grains [2]. The first report of occurrence was from the Indian city Karnal [3] and since then it has been reported from other countries, namely Afghanistan, Iraq, Nepal, Pakistan, Iran, Mexico, Brazil, the United States (New Mexico, Arizona, Texas, and California) and, finally, from South Africa (Northern Cape Province) in the present century [4].

Management of KB has become a serious issue due to various factors such as the pathogen’s dispersal mode, lack of tolerant wheat varieties, and long-term survival of *T. indica* spores in the soil. Therefore, cultural and fungicide control of KB is quite challenging. A compatible coupling of secondary sporidia increases the chance of variability because of heterozygosity. The higher crossover frequency in the pathogen suggested that intraspecific and interspecific hybridization may promote recombination and greater genetic diversity [5]. Variability studies between fungal isolates and virulence are important for disease management and resistance improvement programs. Genetic variation of a pathogen can be understood through the use of genetic markers. Random amplified polymorphic DNA (RAPD), ISSRs, RFLP and AFLP markers and have been confirmed to be useful in finding out the genetic and phylogenetic relationships of organisms [6,7,8]. However, ISSRs and RAPD produce more bands and represent more loci than RFLP and AFLP; and previously 34 RAPD primers and 28 inter-simple sequence repeat (ISSR) markers on 10 isolates collected from different regions of India [8,9]. The mystery of genetic variation in pathogens is widely understood through the use of genetic markers. Diversity of *T. indica* isolates was analyzed using pathological and molecular markers [10,11]. Ref. [12] used ISSR-18, ISSR-1, ISSR-17, ISSR-1, ISSR-19, and RAPD 16 primers on 8 isolates of *T. indica*. Effect of host determinant on variability was determined. They reported both marker systems have indicated the genetic variation between treated and untreated samples of monosporidial strains.

Despite its economic significance, very little work has been done on the molecular component and pathogenesis of the pathogen. In this study, attempts were made to analyze the genetic variations of *T. indica* isolates collected from different districts of Punjab and Khyber Pakhtunkhwa (KPK). More importance was given categorically for precise detection of variation among *T. indica* isolates by using RAPD–ISSR markers. This study, to our knowledge and review the first time a large number of Pakistani isolates has been studied by using molecular techniques of ISSR and RAPD, will explore the links to find out the basis of pathogenicity in *T. indica.*

## 2. Materials and Methods

### 2.1. Sampling and Culturing of KB Isolates

a. Sample collection

**Figure 1:**
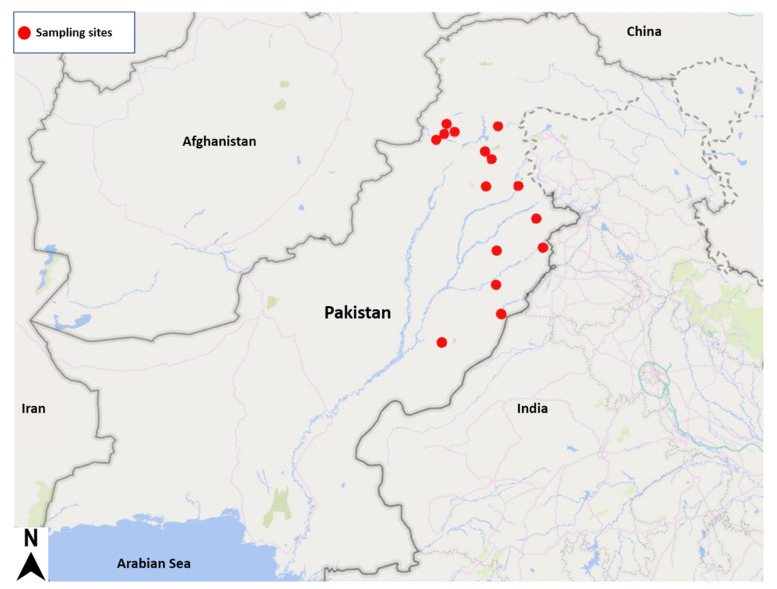
Geographic map of Pakistan showing sampling sites for Karnal bunt (KB) isolates.

Infected wheat grains with KB were randomly collected from Punjab and Khyber Pakhtunkhwa (KP). Surveys were carried out to find the current status of KB during 2013–2014, and 48 isolates were collected [13] (Table 1). Twenty isolates collected during 2010–2011, including Pb-25 (aggressive isolate) [14], were also used for the molecular analysis. The geographic coordinates of the sample collection sites and host cultivars are also given in Table 1. The map positions of geographic coordinates are given in Figure 1.

b. Isolation and multiplication of *T. indica* isolates and fungal inoculation

The infected seeds of 68 isolates (Table 1) containing teliospores were suspended in 10 mL of sterilized distilled water and vortexed for 2 min. The spore suspension was passed through a 60 µm sieve and centrifuged at 12,000 rpm for 2 min. Pellet was suspened in 1% sodium hypochlorite solution and centrifuged for 30 s at 12,000 rpm. Washing of spores with sterilized distilled water was done thrice and for this the spores were first centrifuged at 12,000 rpm for 2 min and then the pellet was re-suspended in sterilized distilled water and plated on the water agar with 1000 µL micro-pipette (Gilson). The plates were incubated at 20 ± 2 °C for 10 days. After 10 days of incubation, the plates were examined under stereomicroscope for the confirmation and morphological studies of the pathogen as described previously. Colonies from single teliospores of *T. indica* were cultured on PDA medium. After isolation of the pure cultures of each isolate of *T. indica*, their mass multiplication for pathogenicity study was done. A small disk of freshly cultured *T. indica* isolate on water agar was cut and placed on the upper edge of flasks/plate containing PDA for further multiplication and inoculum preparation. These flasks were labeled and incubated at 20 ± 2 °C for 15 days. The flasks were stored in the refrigerator to use for further experimentation [15].

c. Pathogen (*T. indica)* material and DNA extraction

As described above in Section b, the mycelia of 68 isolates were collected for DNA extraction, from 15–20-day-old cultures that were incubated at 20 °C. Each mycelial layer of *T. indica* was filtered through autoclaved Whatman filter Paper No. 1 and washed thrice with sterile distilled water. The hyphae mat was dried between sheets of paper at room temperature. The dried mycelia were stored at −20 °C for further use. 

All isolates were cultured separately, multiplied and maintained on Potato Dextrose Agar. The sporidial suspension of each isolate was prepared by adding sterilized distilled water, the fungus was scratched from PDA [16] and the concentration of inoculum was adjusted to 50,000 spores mL^−1^ with a hemocytometer [17].

### 2.2. Pathogenicity Test of KB Isolates 

Eight wheat varieties were selected as these were mega commercially grown varieties in the country, to assess the pathogenicity of isolates, including six commercial wheat varieties, namely, AARI-2011, Bakhtawar-92, Fakhre-Sarhad, Janbaz, NIFA-Barsat, and Tatara, along with 2HD-29 and WL-711 as resistant and susceptible check, respectively. Seeds of wheat varieties were obtained from National Coordinated Wheat Program, National Agriculture Research Centre Islamabad. The wheat varieties were sown in pots, during November 2014. After germination, three plants were maintained per pot. Five heads per pot were inoculated and three inoculated spikes were selected to assess the disease incidence and severity. The experiment was conducted in three replications in a completely randomized design in a two factorials setup.

a. Boot inoculation

The plants were inoculated individually with a spore suspension of each isolate using the boot inoculation method at booting stage [18,19]. Varieties were sown in pots and each variety was replicated thrice in different pots in a glasshouse. Three spikes per variety of each pot were inoculated just before the emergence of awns. Suspension of *T. indica* spores (1mL) from 10–15-day-old cultures was injected into the boot with the aid of a hypodermic syringe. After inoculation, the spikes were labeled and covered with glycine bags to maintain the maximum moisture content for fungus proliferation [20]. The inoculated pots were kept under high humidity (approx. 80%) in the glasshouse, an automated misting system was equipped, and the sprinklers were sprayed five times per day for 20 min each time, under a temperature regime of 18–20 °C [21].

b. Harvesting and scoring

The inoculated spikes were harvested at maturity (58–60 days) after inoculation and threshed manually. The infected seeds were divided into different degrees/grades of infection depending on the extent of the damage on the scoring scale 0–5 [22] (Table 2).

The coefficient of infection (CI) was calculated [23] as follows:

Coefficient of infection= Gross Total/Total numbers of seeds ×100

All isolates of the fungus *T. indica* were selected based on the CI. The isolates were classified as the most aggressive (20.1 and higher), aggressive (10.1–20.0), moderately aggressive (5.1–10.0), slightly aggressive (0.1–5.0) and non-aggressive (0). Similarly, the varieties were categorized on the base of the coefficient of infection value into highly resistant (HR), (0), resistant (R), (0.1–5.0), moderately susceptible (MS), (5.1–10.0), susceptible (S), (10.1–20.0), and highly susceptible (HS), (20.1 and above) 

Percent infection (PI) of each variety was calculated by using the following formula [24,25]:

Percent infection=infected seeds in a sample/Total numbers of seeds in a sample×100

c. Data Analysis

Disease severity of the isolates and varietal response were analyzed based on their CI using Statistix 8.1. Average, percent seed infestation and LSD of both CI and PI were calculated using Statistix software. Design of the experiment was a completely randomized design in two factorials. Analysis of variance as appropriate for the completely randomized design was applied to assess the effect of isolates and varieties on the coefficient of infection and percent seed infestation. The boxplots were prepared in the R-Statistical software.

### 2.3. Genetic Diversity Analysis of KB Isolates

a. DNA extraction

The dried mycelium of *T. indica* (200 mg) of each isolate was taken for DNA extraction using the CTAB method [26] and was allowed to run on 1% agarose gel, along with standard marker for its quantification. The concentration of amplified DNA was assessed visually by checking the band intensity in comparison with Lambda (λ) DNA of known concentration under the UV transilluminator using a gel documentation system. Sharp and separate bands were estimated as good, whereas improper dissolution and thin bands were estimated as sheared [27].

b. Primers used for molecular analysis of *T. indica*

A set of 31 RAPD (Table S1) and 32 ISSR (Table S2) were used for this study. Primers were diluted up to 10 pmol/µL. PCR amplification of *T. indica* isolates was performed in a volume of 20 µL reaction on automated Applied Biosystems Thermal Cycler (Verity 96 well), with composition of 2 µL of 10X buffer, 2 µM of each primer, 0.4 uM dNTPs mixture, 0.2-unit Taq polymerase, 2.4 mM MgCl2, 1ul of template DNA and 12 µL of ddH2O. 

The ISSR amplification regimes were performed at 94 °C for 3 min after that, 35 cycles of 94 °C for 40 s, later annealing was done according to primer for 40 s, followed by extension at 72 °C for 1 min, and a final extension at 72 °C for 10 min. For RAPD amplification, PCR cycles consisted of DNA denaturation at 94 °C for 3 min followed by 37 cycles of 92 °C for 1 min, then annealing was done according to primer for 1 min then extension at 72 °C for 2 min and followed by a final extension at 72 °C for 15 min. 

The amplified DNA or products were run on 1.8% agarose gels at 100 V in 1 × TAE buffer for 1 h along with 1 kb DNA ladder. The DNA band pattern was visualized with UV light and recorded by the imaging system of gel documentation. PCR amplification was repeated thrice to reproduce the DNA profiles with all of the selected primers. A negative control (without DNA template) was added in all of the PCR reactions.

c. Data analysis

Data were computed to evaluate the various parameters for the comparison of RAPD and ISSR primers. The gel of all sets of primers was analyzed. Presence of bands was scored as 1 and absence as 0. A dendrogram was constructed using UPGMA (an unweighted pair method with arithmetic mean) to group individuals into discrete groups [28]. Polymorphic information content (PIC) was calculated as follows:

PIC = 1 − (p/q)^2^ where p is total alleles detected at a given marker locus, q is a collection of genotypes studied. The multiplex ratio (MR) for each primer was assessed by dividing the total number of amplified bands (mono and polymorphic) amplified by the total number of primers (the combination of primers used -n). By using a marker, the average DNA fragments amplified by genotype are known to be a multiplex ratio (n). The number of polymorphic loci within the set of interest of the germplasm analyzed by experiments is called effective multiplex ratio (E) and estimated as E = nβ, where β is a fraction of polymorphic markers. It was calculated by considering the polymorphic loci (np) and a non-polymorphic locus (nnp), as ß = np/ (np + nnp). The usefulness of a given marker system is the balance between the level of polymorphism identified and the extent to which the marker identifies multiple polymorphisms. An informational product measured by PIC and an effective multiplex ratio called the Marker Index (MI) provided a suitable evaluation of the utility of the marker. MI = PIC × E or MI = n × β × PIC.

AMOVA was performed using software R to analyze the genetic diversity of *T. indica* isolates. Escoffier et al. [29] estimated the variance components of the RAPD and ISSR profiles and the assessment of intra- and inter-genetic diversity parameters such as the diversity of the gene Nei (Hexp), Standard error for the rarefaction analysis (SE), Shannon–Wiener Diversity index(H), Stoddard and Taylor’s Index (G), Simpson’s index (Lambda), (E.5)Evenness, Association Index (Ia), Standardized Index of Association for each population factor (rbarD), Observed number of alleles (obs), Standard deviation of data (Std. obs). Genetic structure was studied using the diversity statistics of Nei gene, diversity within population (HS), total genetic diversity (HT), population diversity (D-het) and gene differentiation coefficient (GST) calculated based on the Nei method by using software R [30]. Gene flow estimated from GST was calculated as follows: 

Nm = 0.5 (1-GST)/GST. Principal component analysis (PCA) was performed to obtain a 3D image graphically representing the genetic diversity between the isolates. It was generated using R software [31].

## 3. Results

### 3.1. Variability in Pathogenicity for Isolates of T. indica

Highly significant variability was observed among varieties and isolates for both CI of and PI and their interaction. A total of 49 isolates of *T. indica* (Table 3), isolated from infected wheat seed samples collected from different agro–ecological regions of wheat in 2013–2014, showed variable virulence on different varieties. Forty-six isolates were found to be pathogenic to all varieties of wheat, while three isolates were found non-virulent (Table 3). The disease reaction of forty-nine isolates of *T. indica* on these wheat varieties is shown in Table S3. The highest CI (15.73%) was recorded in isolate PB-25 followed by PB-55 (11.37%) from Faisalabad, while the lowest severity was observed in PB-68, PB-60, and PB-43(10%) collected from Jhelum, Bahawalnagar, and Bahawalpur (Table 3). The mean value of CI showed that isolate PB-25 was aggressive on WL-711 and Fakhre-Sarhad compared to other varieties (Table S3).

**Figure 2:**
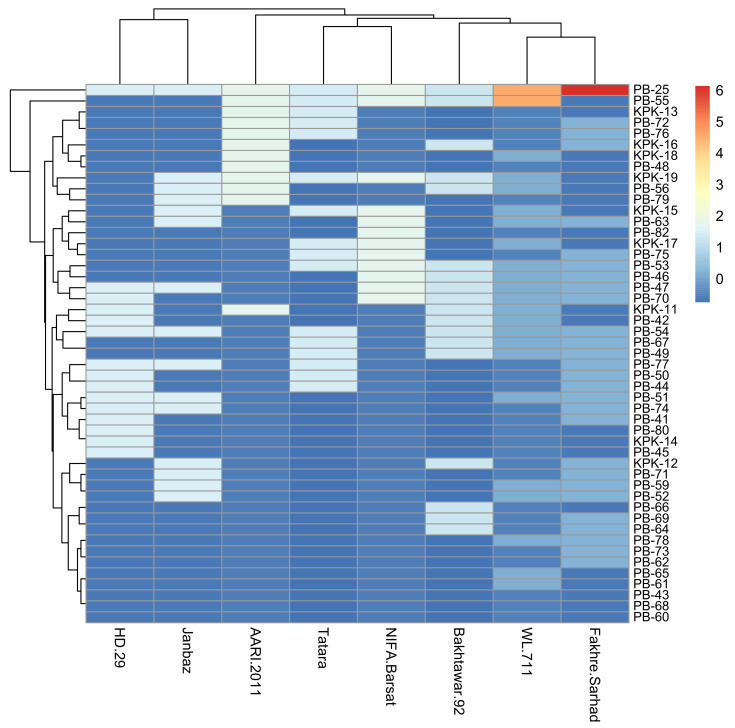
Grouping of forty-nine isolates based on coefficient of infection on eight wheat genotypes.

The aggressive reaction of forty-nine isolates of *T. indica* on these wheat varieties was subjected to analysis of variance (ANOVA). The results revealed that there was a highly significant difference of isolates among themselves and their reaction on different varieties tested at *p* ≤ 0.01. To clarify the aggressiveness behavior of forty-nine isolates of *T. indica* based on their reaction on eight wheat varieties, the results on these varieties were subjected to cluster analysis using Minitab16. The dendrogram (Figure 2) is based on the combined means of the coefficient of infection of *T. indica* on varieties. Based on aggressiveness behavior, the isolates were clustered into different groups and subgroups, namely Group I (subgroup = 0), Group II (subgroup = IIA, IIB), Group III (subgroup IIIA and IIIB). Group I comprised one isolate (PB-25) and belonged to a moderately aggressive category. Group II consisted of slightly aggressive isolates having a single isolate in each subgroup IIA, IIB. Group IIIA comprised three non-aggressive isolates, while Group III B comprised forty-three isolates and belonged to weakly aggressive category. Although the two groups (Group II, subgroups II A and II B, and Group III subgroup III B) were based on CI, they belonged to a weakly aggressive category, but based on cluster analysis and similarity these comprised different groups (Figure 2).

### 3.2. Reactions of Wheat Varieties against KB Isolates

Eight varieties, including one resistant and susceptible check, were tested for their responses against 49 isolates of *T. indica.* WL-711 exhibited moderately susceptible response and showed a maximum CI value (10.88%) (Table 4). The varieties NIFA-Barasat, HD-29, Janbaz, Bakhtawar-92, Tatara, and AARI 2011 showed a resistant to highly resistant response to all the isolates. Fakhre-Sarhad showed a moderately susceptible to highly resistant response (Table S3).

Data for mean percent seed infected with KB disease for each treatment was calculated. The data were subjected to ANOVA. The results revealed that there was a highly significant effect of isolates among themselves and their reaction on different varieties tested at *p* ≤ 0.01. The isolate PB-25 (32.93%) caused the highest PI on all the wheat varieties tested followed by PB-55 (17.99%), whereas the isolate PB-64 (10.29%) exhibited the least infection. The three isolates PB- 43, 68, and 60 were found to be nonpathogenic on these varieties (Table 3). Comparison of the overall distribution of CI values and PI revealed a strong correlation between the two parameters, with an R^2^ value of 0.764.

### 3.3. Variability in T. indica Percent Infection as a Function of Sampling Location

**Figure 3:**
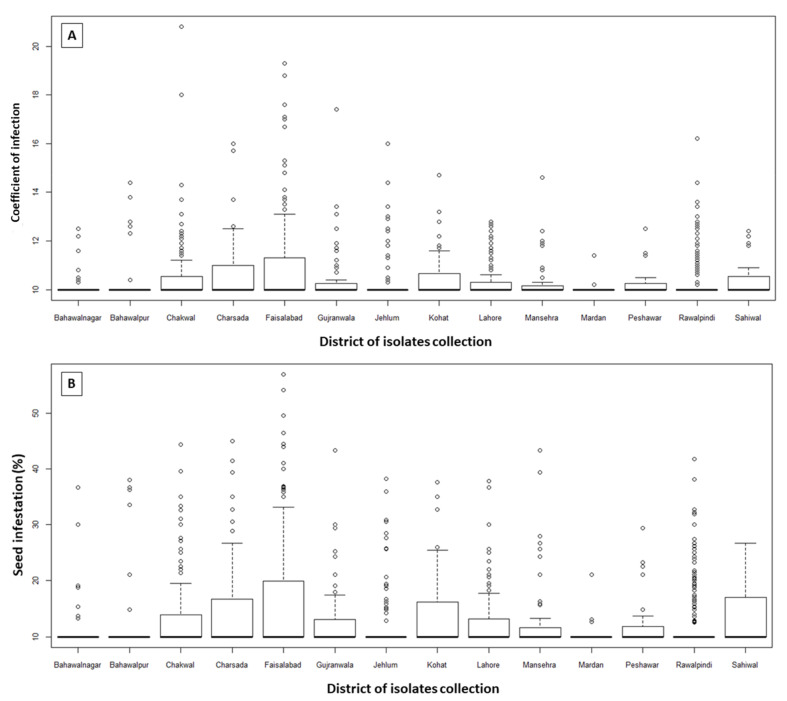
Variability in Karnal bunt coefficient of infection (**A**) and percent infection (**B**) for *T. indica* isolates sampled from different districts of diverse wheat growing regions of Pakistan.

Analyzing the response of *T. indica* isolates, while considering their location of sampling, revealed substantial variability in their pathogenicity as assessed by the coefficient of infection and percent infection (Figure 3). The result revealed that based on the sampling from different geological areas the variability significantly exists in the isolates. Isolates sampled from Faisalabad had the maximum variability for percent infection of seeds, followed by samples collected from Rawalpindi, Charsada, Chakwal and Gujranwala. Across the studied major wheat growing regions of Pakistan, the samples collected from southern Punjab had the maximum percent infection (with a mean value of 14.13) followed by Khyber Pakhtunkhwa (Table 4). A similar pattern was observed for CI, the maximum observed at district Faisalabad of Punjab (13.7), while the minimum (10.0) was observed for isolates sampled from Northern Punjab district Jehlum and Rawalpindi, and southern Punjab district of Bahawalnagar (Figure 3).

### 3.4. Banding Patterns of RAPD and ISSR Primers

Thirty-one RAPD primers (Table S1) were selected for this study based on their reproducibility, the number of polymorphic fragments per primer and the level of polymorphism detected in a particular population. Eight primers were selected. A total of 99 different bands were amplified and found to be (100%) polymorphic. Bands amplified per primer ranged from 9 (OPA-9) to 15 (OPA-20, RAPD-16) with an average of 12.37. The size of the product ranged between 0.25 kb and 2.5 kb. Four unique bands were found: OPA 3 (on isolate 17), OPA 18 (isolate 61), and OPA 20 (isolates 15 and 30). The average PIC values, is a description of allele diversity and frequency, found in this study among the *T. indica* species were 0.93, ranging from 0.8 (RAPD 16) to 0.98 (OPA 20) (Table 5).

Among thirty-two ISSR primers (Table S2) tested, 20 were useful for characterizing samples, while 11 were excluded due to the absence of amplification. The 20 selected ISSR primers produced 215 bands, 100% of which were polymorphic with an average of 10.75 bands per primer. Bands produced by each of the twenty ISSR ranged from 6 (ISSR-1) to 15 (876 and 856-2). The size of the product ranged from 0.25–3.0 kb. Nine unique bands were produced from the primers 812(15, 58), ISSR-1(41), 845(38, 44), 847 (46), 817 (15), 856 (1), and 856-2(17). Values in parentheses denote the code number assigned to the isolates as sequenced in Table 6. The average PIC values of the *T. indica* species used in this study 0.902 ranged from 0.69 (ISSR 17) to 0.973 (ISSR-18) (Table 6).

#### 3.4.1. Analysis of Molecular Variance (AMOVA)

AMOVA analysis among and within isolates of *T. indica* under study was performed to test the population differentiation utilizing molecular markers. For the analysis of molecular variance based on RAPD, 7.76% diversity was computed among the population, whereas variations within the isolates were 90.44%. ISSR analysis showed 7.408% variability among the population and 91.09% within the isolates. Genetic diversity between the isolates and among the population of *T. indica* was statistically significant (Table 7).

#### 3.4.2. Principal Component Analysis (PCA) of RAPD and ISSR

**Figure 4:**
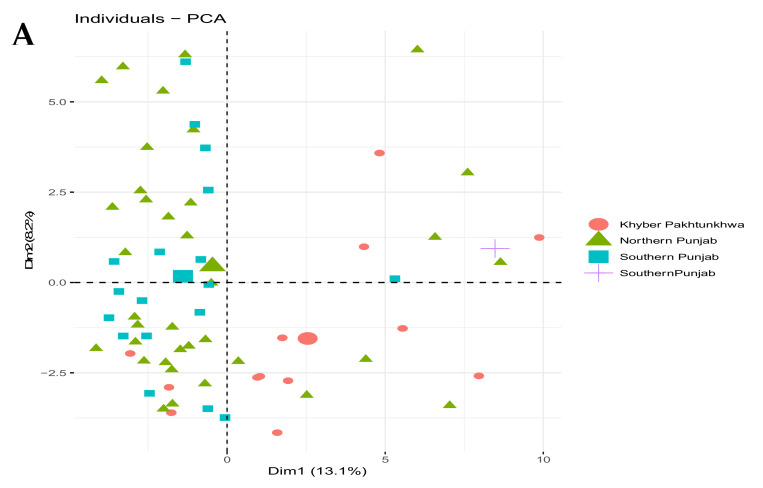

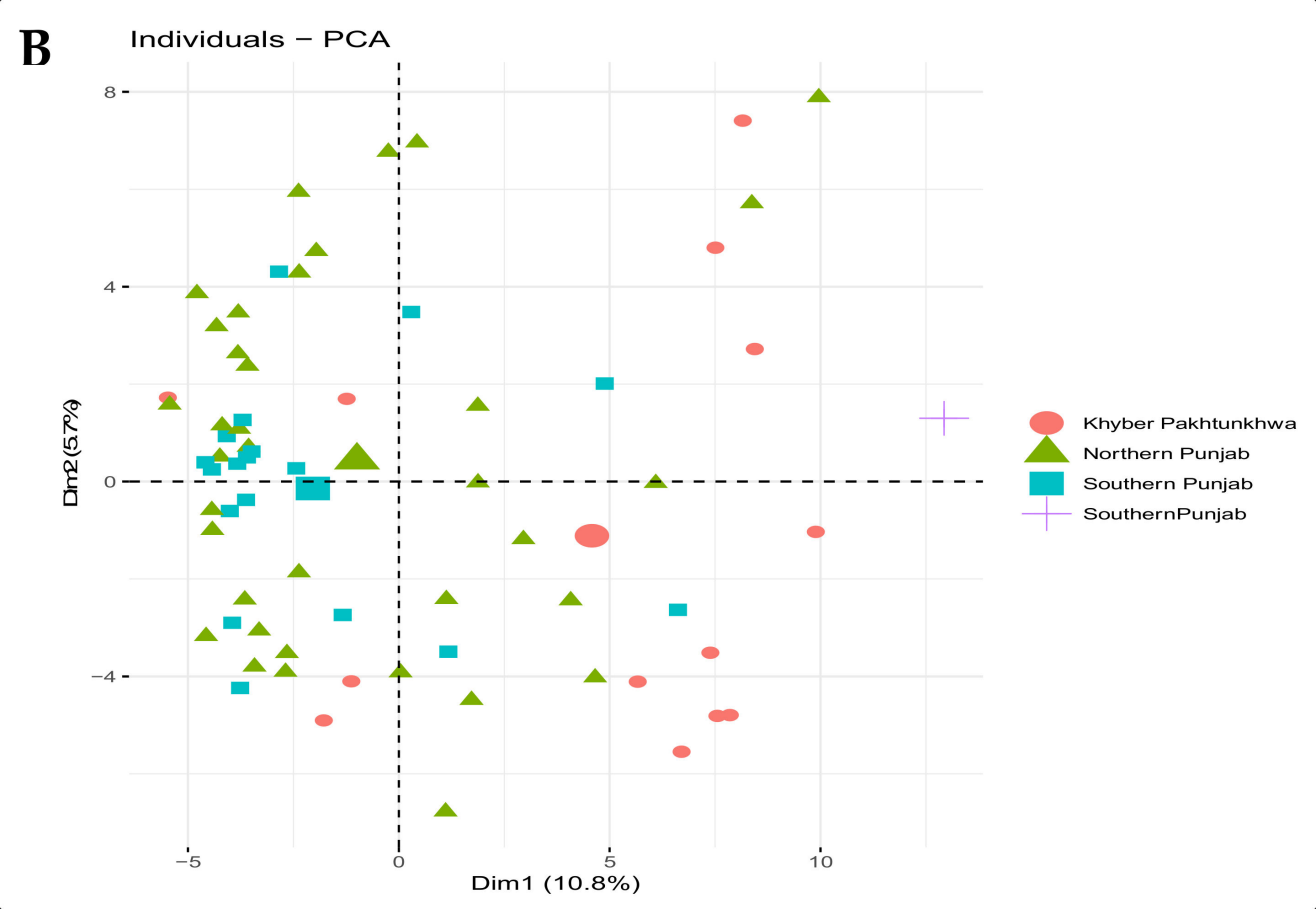
Distribution of *T. indica* isolates revealed by principal component analysis (PCA) based on (**A**) RAPD and (**B**) ISSR.

The principal component analysis was done to verify the grouping of isolates based on RAPD and ISSR. The first three Eigenvectors showed the minimum range of the data and accounted for 30% of the total variance in RAPD, 23.23% in ISSR, whereas the first component accounted 14.89% and 11.77% variance, respectively (Table S4). Two-dimensional (2D) distributions of the *T. indica* isolates based on PCA values in RAPD and ISSR analysis gave a perfect discrepancy of the sixty-eight *T. indica* isolates used in the study (Figure 4A). PCA plot of RAPD and ISSR showed that the groups (Punjab and KPK) are intersecting but are different (Figure 4B).

#### 3.4.3. Combined RAPD and ISSR Cluster Analysis

**Figure 5:**
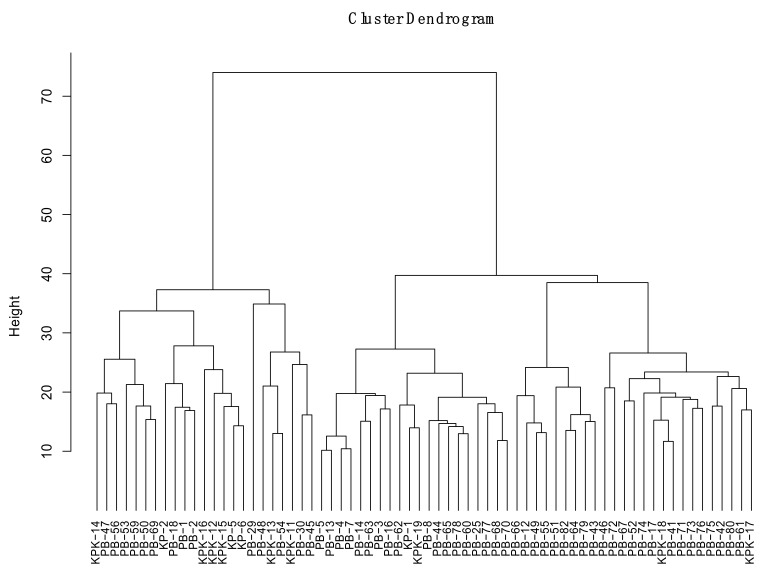
Dendrogram constructed by using UPGMA based on the Euclidean coefficient of combined RAPD–ISSR for 68 *T. indica* isolates.

Euclidean similarity coefficient was calculated using the combined 8 RAPD and 20 ISSR primers data. Similarity coefficient (Sm) values ranged from 0.4 to 0.9 in combined RAPD and ISSR analysis. UPGMA cluster analysis grouped the isolates of *T. indica* into three major groups (I, II and III) that were further subdivided into two subgroups each. Group, I A comprised one isolate, while I B comprised 9 isolates. Group II A comprised two isolates, whereas Group II B comprised 22 isolates. Group III A comprised thirteen isolates and Group III B comprised 21 isolates (Figure 5).

#### 3.4.4. Genetic Diversity in *T. indica* Isolates

**Figure 6:**
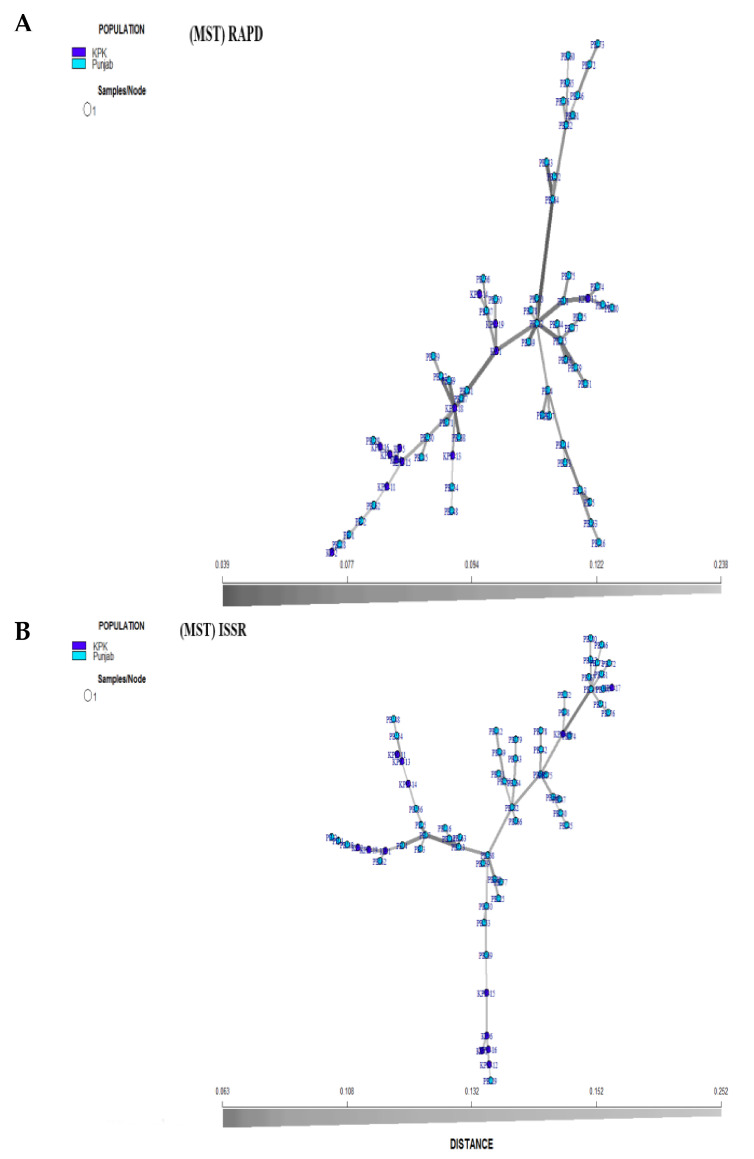
Minimum Spanning Tree (MST) analysis using Bruvo’s distance illustrating the relationships and the relative frequency of each isolates. Circle colors represent the population of *T. indica* isolates based on (**A**) RAPD, (**B**) ISSR.

Genetic diversity of *T. indica* isolates based on RAPD and ISSR was calculated. In the analysis of RAPD and ISSR, isolates from two provinces (Punjab, KPK) of Pakistan were evaluated. Among 68 isolates, 55 isolates from Punjab and 13 from KPK were evaluated in this study and all were multilocus (MLG). Expected MLG based on rarefaction was 13 for both RAPD and ISSR analysis (Table 8). The minimum spanning tree for the frequency of isolates for RAPD and ISSR markers using Bruvo’s distance was formed (Figure 6A,B). The Shannon–wiener index (H) and MLG diversity (G) in RAPD were 4.01 and 55 for Punjab, whereas in the case of ISSR it was 2.56 and 13 in KPK population, respectively. Simpson’s index (lambda) was estimated (0.98) and (0.923) in Punjab and KPK, respectively, and found to be same for all sets of markers. Evenness was 1 and showed that genotypes were fairly evenly distributed. Evenness of Punjab and KPK isolates (1) was similar in RAPD, ISSR. The Nei’s gene diversity ranged from 0.2 to 0.209 in Punjab and 0.204 to 0.242 in KPK, and was detected by the RAPD, ISSR, (0.2, 0.209) respectively. Test for multilocus association (IA) was significantly different from zero (*p* < 0.001), indicating a linkage disequilibrium in all populations (Table 8). Ia values for RAPD (5.32) and ISSR (5.59) in the isolates collected from Punjab and association of KPK samples were 5.2 for RAPD and 3.83 for ISSR. Standardized index of association ranged from 0.0179 to 0.0683 and showed the maximum value in RAPD (0.0374) as compared to ISSR (0.0179 in Punjab, and showed the maximum value in RAPD (0.0452) as compared to ISSR (0.0149) in KPK (Table 8).

#### 3.4.5. Genetic Structure and Gene Flow in *T. indica* Isolates

Total population diversity (HT), within population diversity (Hs), diversity between the population (D-het) and Gene flow (Nm) were calculated by using R software. HT (total diversity for among the population) was higher in ISSR (0.23), while the lowest diversity was found in RAPD (0.21). The highest value of HS was found in ISSR 0.22 and (0.19) in RAPD. The mean coefficient of gene differentiation (GST-est and G prime-st) was higher in RAPD (0.05, 0.13) than ISSR (0.05, 0.12), respectively. Gene flow was relatively higher in ISSR (9.69). The diversity between the populations (D-het) was higher in ISSR (0.029) (Table S5).

#### 3.4.6. Comparison of RAPD and ISSR in Evaluating Genetic Diversity of *T. indica*

The marker systems (RAPD and ISSR) were compared according to different criteria (Table 9). Detection of polymorphism, unique band, means PIC value, MR, EMR, and MI. Analysis by RAPD and ISSR showed a polymorphism level of 100%. In the present study, the average number of DNA fragments amplified by RAPD and ISSR was 12.37 and 10.75, respectively. This high variation produced in the number of fragments by these arbitrary primers can be ascribed to differences in the binding sites along the genome of the T. indica isolates used. The PIC value of the RAPD primers was higher (0.92). The multiple ratio (MR), effective multiplex ratio (EMR) and marker index (MI) in RAPD (Table 9) were higher than ISSR. The RAPD and ISSR data were combined for UPGMA cluster analysis. The combined cluster of RAPD and ISSR was 80-85%, similar to each of the separate (RAPD or/ISSR) cluster analyses (Figure 6).

## 4. Discussion

Compared to other sumt fungi, the control of KB is challenging due to its heterothallic nature and sporadic occurrence [22]. Unfortunately, no work was previously conducted to understand and explore the pathogenicity and genetic variability of *T. indica* in Pakistan. However, limited studies on the variability of *T. indica* in terms of size of primary and secondary sporida, germination percent, morphology [13,15,32] host reaction, and aggressiveness among isolates in pathogenicity tests were reported earlier from India and Pakistan [14,33,34].

Our results revealed significantly variable pathogenicity in *T. indica* isolates collected from different locations of Pakistan in terms of CI and PI (%). The isolate PB-25 reported earlier was also found significantly pathogenic as compared to other isolates assessed in the current study (Table 2). Among other isolates, PB-55 has an aggressive response and was collected from the same district as the reference isolate [14].

Based on pathogenic behavior, the isolates were arranged into three major groups. Group I contain moderately aggressive isolate (PB-25), Group II contained weakly aggressive isolates, and Group III comprised weakly and non-aggressive isolates (Figure 2). Grouping of isolates into various clusters was based on their response to host [16,35]. The clustering was independent of the location of sampling for the given isolate. This would imply that variable pathogenicity exists among the isolates prevalent at a given location in major wheat-growing regions of Pakistan. Previously, similar results of lack of location-specific virulence variability were reported for *Puccinia striiformis* populations from Pakistan [36]. The prevalence of aggressive isolates has been suggested to be present in southern Punjab, Pakistan [37]. However, our findings revealed the prevalence of the aggressive isolate from Northern Punjab (Table 3), which is an indication of shifting of the pathogen from one area to another area. This has proven to be an alarming situation for the country as the area belongs to the major wheat-growing zones of the country.

Considering the airborne and seed-borne dispersal of the disease [38], the hot spot of KB could be shifting from one place to another over the seasons. This will require a more collaborative effort while considering the deployment of varieties and disease management strategies. The situation could further be complicated by the assortment of virulence genes during sexual reproduction of the pathogen, which leads to the breakdown of host races [10,39] since the life cycle of *T. Indica* differs from those of other bunt diseases of wheat in several ways as they germinate and produce haploid primary sporidia (basidiospores) that conjugate immediately on the promycelium and then produce dikaryotic infectious hyphae or dikaryotic secondary sporidia that systemically infect seedling plants. In contrast, teliospores of *T. indica* germinate and produce monokaryotic, haploid, and primary sporidia that do not conjugate, but rather germinate and produce numerous monokaryotic, haploid, and secondary sporidia. The secondary sporidia are airborne to plant surfaces where they may germinate and produce additional generations of secondary sporidia [2,8,33]. Further, because of the heterothallism and the complex phenomenon of race existence, the varieties needed to be tested against multiple isolates for their pathogenicity [25,40,41].

The reaction of the eight varieties against 49 isolates of *T. indica* revealed WL-711 exhibited a moderately susceptible response. The varieties NIFA-Barasat and HD-29, Janbaz, Bakhtawar-92, Tatara, and AARI -2011 showed a resistant to highly resistant response against all the isolates. The variety Fakhre-Sarhad showed a moderately susceptible to highly resistant response (Table S3). These findings show that these varieties may possess multiple disease-resistance genes [14,42] and reported a resistant to a high-resistant reaction in varieties such as NIFA-Barasat, Punjab-2011, BARA-09 and Seher against a set of 21 *T. indica* isolates. The significant effect of isolates and varieties suggested the presence of pathogenic variation in *T. indica* isolates as well as the resistance genes in these cultivars [34], which could result from the gene-to-gene interaction dependent on a strain-host relationship [37].

There are several field experimental studies to assess the response of varieties to natural infection of KB; however, limited studies have been done to assess the variability in a large number of pathogen isolates in terms of aggressiveness. Although such studies are regularly done for rust pathogens, fewer report on KB. The pathogenic variability observed in many isolates is helpful in disease-resistance development and deployment [43,44]. The pathogen adaptation potential is directly linked with the pathogen isolate diversity, which enables the acquisition of virulence against the deployed host resistance [45]. The study must be conducted across multiple years to track the pathogen evolution in time. The isolates must also be compared with other regional partners in India, Afghanistan, and Iran to understand the potential of the pathogen in the context of invasions, as revealed in another wheat pathogen such as rusts. Such studies on pathogenic variability must thus enable better development and deployment of KB tolerance and resistance at the farmer field.

*T. indica* isolates were found to be highly diverse even in single infected grain [46]. Previously, the variability of *T. indica* was analyzed by using RAPD and ISSR on a very limited number of isolates. RAPD and ISSR techniques generated numerous polymorphic bands, and the presence of a high percentage of polymorphism indicated high variability in this pathogen targeted by the primers [9]; however, in our study RAPD and ISSR primers generated 100 percent polymorphic bands, and no monomorphic band was found in each set of primers. Similar results were observed by other scientists who reported that DNA markers such as RAPD and ISSR [9,47,48] are useful for detecting, differentiating, and determining phylogenetic relationships between isolates of morphological species of different pathogens [7,8,49].

In this study, some unique bands of RAPD (4) and ISSR (9) were obtained. These unique bands were special to a particular isolate, making them distinguishable from others. These unique bands can be used as markers to identify the important isolates. Parveen et al. [9] also observed a similar pattern in banding profiles obtained from RAPD and ISSR markers. In our study results of AMOVA based on ISSR and RAPD marker analyses showed the genetic variation of *T. indica* isolates under study. Significant variability was found in gene diversity among the population and within the isolates at p-value 0.05. Although the difference between the isolates was not significant, this was due to the small geographical distance of the sample collection sites for the isolated pathogen. ISSR-based AMOVA reveals the highest differences of *T. indica* isolates when comparing variations within species. However, in another study conducted by Medhi et al. [50] on *zanthoxylum* spp., they reported high variation in RAPD markers in AMOVA analysis.

Standardized index of association of Punjab and KPK showed the maximum value in RAPD (0.034 and 0.045), respectively, in the whole population. The tests for multilocus association (IA) were all significantly different from zero (*p* < 0.001), revealing that there is linkage disequilibrium in all populations. Genetic diversity within a species is often associated with geographic extent, reproductive patterns, coupling systems, and seed dispersion and reproduction [51]. Genetic structure and gene flow of the *T. indica* isolates were found by the mean of the parameters such as total and within population diversity, coefficient of gene differentiation, and gene flow. Total (HT) and within population (HS) diversity were found to be the highest in ISSR with the high gene flow compared to RAPD. Coefficient of gene differentiation was higher in RAPD. In population genetics, the value of gene flow (Nm) ≤ 1.0 (less than one migrator per population in the population) or equivalently, the gene differentiation value (GST) > 0.25 is generally considered a threshold [52]. Genetic flow values above 1 are “strong enough” to avoid significant differences due to genetic drift. The extension of genetic variation in species always favors selection (drift, gene flow) and nonselectivity (natural selection) in population segmentation. Taking into account these criteria, the *T. indica* population have exceeded the threshold level, and ISSR is excellent in gene flow and gene differentiation assessment (ISSR (Nm = 9.6, GST = 0.05) in comparison to RAPD (Nm = 8.84, GST = 0.05). This information generated on such a large number of isolates of *T.indica* from diversified ecological zones of Pakistan to assess genetic diversity is a first attempt that could be helpful for not only the breeders but the pathologists of South East Asia in combating this quarantine significance disease by exploring appropriate links for resistance genes.

## 5. Conclusions

Knowledge of the aggressiveness, pathogenicity, and diversity of the *T. indica* is essential for effective management of the KB disease. The pathogenicity of a countrywide collection of KB isolate was resolved, which indicated more aggressive isolates from Punjab compared to the KPK part of Pakistan. Both the molecular marker systems, RAPD and ISSR, further supported the pathogenicity by similar patterns of genetic diversity. The molecular markers identified the genetic diversity and differentiated the isolates based on geographic prevalence and host cultivar. The isolates from Punjab were more genetically diverse compared to the isolates from KPK.

## Figures and Tables

**Table 1:** Isolates of *T. indica* collected form Punjab and Khyber Pakhtunkhwa (KPK) during 2010–2011 and 2013–2014.

Isolate Name	Year	Populations	District	Host Cultivar	Latitude	Longitude
KP-1	2010–2011	Khyber Pakhtunkhwa	Peshawar	Unknown	34.0151	71.5249
KP-2	2010–2011	Khyber Pakhtunkhwa	Charsada	Unknown	34.14943	71.74278
KP-5	2010–2011	Khyber Pakhtunkhwa	Mardan	Unknown	34.1989	72.0231
KP-6	2010–2011	Khyber Pakhtunkhwa	Mardan	Unknown	34.1989	72.0231
KPK-11	2013–2014	Khyber Pakhtunkhwa	Charsada	Fsd-2008	34.14943	71.74278
KPK-12	2013–2014	Khyber Pakhtunkhwa	Peshwar	BARS-09	34.0151	71.5249
KPK-13	2013–2014	Khyber Pakhtunkhwa	Kohat	Pirshabak	34.385	71.806
KPK-14	2013–2014	Khyber Pakhtunkhwa	Mardan	Sehr-2008	34.1989	72.0231
KPK-15	2013–2014	Khyber Pakhtunkhwa	Charsada	Sehr	34.14943	71.74278
KPK-16	2013–2014	Khyber Pakhtunkhwa	Charsada	Uqab	34.14943	71.74278
KPK-17	2013–2014	Khyber Pakhtunkhwa	Mansehra	Bakhar	34.33126	73.198
KPK-18	2013–2014	Khyber Pakhtunkhwa	Mansehra	Fakhar-e- Sarhad	34.33126	73.198
KPK-19	2013–2014	Khyber Pakhtunkhwa	Kohat	Glaxay	34.385	71.806
PB-1	2010–2011	Northern_ Punjab	Chakwal	BARS-2009	32.9328	72.863
PB-12	2010–2011	Southern_ Punjab	Sahiwal	FD-4	30.6682	73.1114
PB-13	2010–2011	Southern_ Punjab	Sahiwal	Watan	30.6682	73.1114
PB-14	2010–2011	Southern_ Punjab	Sahiwal	INQ-91	30.6682	73.1114
PB-16	2010–2011	Southern_ Punjab	Bahawalnagar	Aas-2011	30.0025	73.2412
PB-17	2010–2011	Southern_ Punjab	Bahawalnagar	Sehr-2006	30.0025	73.2412
PB-18	2010–2011	Southern_ Punjab	Bahawalnagar	Unknown	30.0025	73.2412
PB-2	2010–2011	Northern_ Punjab	Jhelum	Sehr-2006	32.9425	73.7257
PB-25	2010–2011	Southern_ Punjab	Faisalabad	Unknown	31.4504	73.135
PB-29	2010–2011	Southern_ Punjab	Bahawalpur	Faisalabad-08	29.3544	71.6911
PB-3	2010–2011	Northern_ Punjab	Rawalpindi	Pak-81	33.7463	72.8397
PB-30	2010–2011	Southern_ Punjab	Bahawalpur	INQ-91	29.3544	71.6911
PB-4	2010–2011	Northern_ Punjab	Rawalpindi	Bakhtawar	33.5651	73.0169
PB-41	2013–2014	Northern_ Punjab	Rawalpindi	Chakwal-96	33.5651	73.0169
PB-42	2013–2014	Southern_ Punjab	Bahawalpur	Millat-2011	29.3544	71.6911
PB-43	2013–2014	Southern_ Punjab	Bahawalpur	Shahensha	29.3544	71.6911
PB-44	2013–2014	Northern_ Punjab	Rawalpindi	Sehroz	33.5651	73.0169
PB-45	2013–2014	Northern_ Punjab	Rawalpindi	Nayab-81	33.5651	73.0169
PB-46	2013–2014	Northern_ Punjab	Rawalpindi	Blue Silver	33.5651	73.0169
PB-47	2013–2014	Northern_ Punjab	Chakwal	Koh-e- noor	32.9328	72.863
PB-48	2013–2014	Northern_ Punjab	Chakwal	Lasani	32.9328	72.863
PB-49	2013–2014	Southern_ Punjab	Sahiwal	Dharabi	30.6682	73.1114
PB-5	2010–2011	Northern_ Punjab	Taxila	Shehzor-2007	33.7463	72.8397
PB-50	2013–2014	Northern_ Punjab	Chakwal	Chakwal-50	32.9328	72.863
PB-51	2013–2014	Northern_ Punjab	Chakwal	Margala	32.9328	72.863
PB-52	2013–2014	Southern_ Punjab	Faisalabad	Fsd-85	31.4504	73.135
PB-53	2013–2014	Northern_ Punjab	Chakwal	C-518	32.9328	72.863
PB-54	2013–2014	Northern_ Punjab	Jehlum	C-591	32.9425	73.7257
PB-55	2013–2014	Southern_ Punjab	Faisalabad	Pak-81	31.4504	73.135
PB-56	2013–2014	Northern_ Punjab	Chakwal	Miraj-2008	32.9328	72.863
PB-59	2013–2014	Southern_ Punjab	Bahawalnagar	Chakwal-50	30.0025	73.2412
PB-60	2013–2014	Southern_ Punjab	Bahawalnagar	GA-2002	30.0025	73.2412
PB-61	2013–2014	Northern_ Punjab	Rawalpindi	Sehzor-2007	33.7463	72.8397
PB-62	2013–2014	Northern_ Punjab	Rawalpindi	Sehr-08	33.7463	72.8397
PB-63	2013–2014	Northern_ Punjab	Rawalpindi	Baras-2009	33.7463	72.8397
PB-64	2013–2014	Southern_ Punjab	Bahawalnagar	Pirsabak	30.0025	73.2412
PB-65	2013–2014	Northern_ Punjab	Jehlum	Unknown	32.9425	73.7257
PB-66	2013–2014	Northern_ Punjab	Jehlum	Aas	32.9425	73.7257
PB-67	2013–2014	Northern_ Punjab	Jehlum	Shafaq	32.9425	73.7257
PB-68	2013–2014	Northern_ Punjab	Jehlum	Chakwal-50	32.9425	73.7257
PB-69	2013–2014	Northern_ Punjab	Lahore	Unknown	31.5204	74.3587
PB-7	2010–2011	Northern_ Punjab	Rawalpindi	Seher-2006	33.5651	73.0169
PB-70	2013–2014	Northern_ Punjab	Lahore	Pirsabak	31.5204	74.3587
PB-71	2013–2014	Northern_ Punjab	Lahore	Pak-81	31.5204	74.3587
PB-72	2013–2014	Northern_ Punjab	Lahore	Unknown	31.5204	74.3587
PB-73	2013–2014	Northern_ Punjab	Gujrawala	Sehr-08	32.1877	74.1945
PB-74	2013–2014	Northern_ Punjab	Gujrawala	Pak-81	32.1877	74.1945
PB-75	2013–2014	Northern_ Punjab	Gujrawala	Miraj	32.1877	74.1945
PB-76	2013–2014	Northern_ Punjab	Rawalpindi	Sehzor-2007	33.5651	73.0169
PB-77	2013–2014	Northern_ Punjab	Rawalpindi	Uqab-2000	33.5651	73.0169
PB-78	2013–2014	Northern_ Punjab	Rawalpindi	Sehr 07	33.5651	73.0169
PB-79	2013–2014	Northern_ Punjab	Rawalpindi	Watan	33.5651	73.0169
PB-8	2010–2011	Northern_ Punjab	Jhelum	Pak- 81	32.9425	73.7257
PB-80	2013–2014	Southern_ Punjab	Faisalabad	Chakora	31.4504	73.135
PB-82	2013–2014	Southern_ Punjab	Faisalabad	Ilasafi	31.4504	73.135

**Table 2:** Rating scale used to assess wheat cultivars for Karnal bunt (*Tilletia indica*).

Infection Category/Grade	Symptoms	Numerical Value for Calculation of (CI)
0	Healthy	0
1 *	Inconspicuous point infection (trace) 5% seed bunted	0.25 *
2 *	Well-developed point infection 25% seed bunted	0.25 *
3	Infection spreading along the groove 50% seed bunted	0.5
4	Three-quarters of seed converted to sorus 75% seed bunted	0.75
5	Seed completely converted to sorus 100% seed bunted	1.0

* Categories combined to calculate Coefficient of Infections for comparison.

**Table 3:** The aggressiveness behavior of *T. indica* on different wheat varieties.

District	Isolate Code	Population	PI (%) ± SD	CI ± SD *	Reaction Based on CI
Charsada	KPK-11	KPK	15.4 ± 8.12	10.77 ± 1.34	SA
	KPK-15	KPK	14.725 ± 8.32	10.65 ± 1.34	SA
	KPK-16	KPK	15.6 ± 10.28	10.59 ± 1.05	SA
Kohat	KPK-13	KPK	11.53 ± 4.34	10.23 ± 0.68	SA
	KPK-19	KPK	16.53 ± 8.94	10.88 ± 1.3	SA
Mansehra	KPK-17	KPK	15.464 ± 9.76	10.62 ± 1.14	SA
	KPK-18	KPK	11.32 ± 3.76	10.19 ± 0.55	SA
Mardan	KPK-14	KPK	10.71 ± 2.36	10.08 ± 0.3	SA
Peshawar	KPK-12	KPK	12.7 ± 5.5	10.33 ± 0.68	SA
Chakwal	PB-47	Northern Punjab	14.464 ± 6.31	10.55 ± 0.8	SA
	PB-48	Northern Punjab	11.24 ± 6.05	10.45 ± 2.2	SA
	PB-50	Northern Punjab	12.21 ± 4.07	10.31 ± 0.68	SA
	PB-51	Northern Punjab	15.24 ± 8.88	10.82 ± 1.8	SA
	PB-53	Northern Punjab	13.24 ± 5.97	10.67 ± 0.94	SA
	PB-56	Northern Punjab	13.49 ± 6.99	10.48 ± 1.01	SA
Gujranwala	PB-73	Northern Punjab	12.29 ± 7.35	10.43 ± 1.54	SA
	PB-74	Northern Punjab	11.82 ± 3.21	10.23 ± 0.45	SA
	PB-75	Northern Punjab	14.12 ± 6.64	10.62 ± 1.06	SA
Jehlum	PB-54	Northern Punjab	13.89 ± 6.92	10.43 ± 0.81	SA
	PB-65	Northern Punjab	12.49 ± 7.21	10.41 ± 1.33	SA
	PB-66	Northern Punjab	10.86 ± 2.4	10.108 ± 0.32	SA
	PB-67	Northern Punjab	13.89 ± 6.96	10.78 ± 1.29	SA
	PB-68	Northern Punjab	10 ± 0	10 ± 0	NA
Lahore	PB-69	Northern Punjab	11.071 ± 2.73	10.12 ± 0.29	SA
	PB-70	Northern Punjab	14.34 ± 6.69	10.53 ± 0.83	SA
	PB-71	Northern Punjab	12.02 ± 4.35	10.33 ± 0.78	SA
	PB-72	Northern Punjab	13.15 ± 7.05	10.45 ± 0.9	SA
Rawalpindi	PB-41	Northern Punjab	10.73 ± 2.46	10.31 ± 0.91	SA
	PB-44	Northern Punjab	11.51 ± 3.55	10.17 ± 0.37	SA
	PB-45	Northern Punjab	10.708 ± 2.52	10.07 ± 0.25	SA
	PB-46	Northern Punjab	14.68 ± 8.29	10.55 ± 0.95	SA
	PB-61	Northern Punjab	11.186 ± 4.66	10.3 ± 1.27	SA
	PB-62	Northern Punjab	10.39 ± 1.45	10.041 ± 0.16	SA
	PB-63	Northern Punjab	12.76 ± 6.55	10.54 ± 0.99	SA
	PB-76	Northern Punjab	13.35 ± 8.18	10.43 ± 0.83	SA
	PB-77	Northern Punjab	14.53 ± 5.06	10.46 ± 0.7	SA
	PB-78	Northern Punjab	12.99 ± 3.95	10.31 ± 0.68	SA
	PB-79	Northern Punjab	11.65 ± 4.37	10.31 ± 1.05	SA
Faisalabad	PB-25	Northern Punjab	31.676 ± 9.92	15.73 ± 2.47	MA
	PB-52	Northern Punjab	12.09 ± 3.46	10.31 ± 0.55	SA
	PB-55	Northern Punjab	17.99 ± 12.75	11.37 ± 2.09	SA
	PB-80	Northern Punjab	10.45 ± 1.54	10.043 ± 0.15	SA
	PB-82	Northern Punjab	10.48 ± 1.69	10.053 ± 0.2	SA
Bahawalnagar	PB-59	Southern Punjab	12.92 ± 6.91	10.32 ± 0.73	SA
	PB-60	Southern Punjab	10 ± 0	10 ± 0	NA
	PB-64	Southern Punjab	10.29 ± 0.99	10.027 ± 0.09	SA
Bahawalpur	PB-42	Southern Punjab	15.011 ± 9.95	10.68 ± 1.36	SA
	PB-43	Southern Punjab	10 ± 0	10 ± 0	NA
Sahiwal	PB-49	Southern Punjab	13.24 ± 5.53	10.43 ± 0.8	SA
	LSD (0.01)		0.625	3.54	

* Transformed data by adding 10. PI = Percent infection (%), CI = Coefficient of infection, MA = moderately aggressive, SA = Slightly aggressive, NA = non-aggressive.

**Table 4:** Aggressiveness analysis of *T. indica* isolates on wheat varieties based on coefficient of infection and percent infection, as expressed against isolates from various wheat growing regions.

Wheat Variety	Khyber Pakhtunkhwa		Northern Punjab		Southern Punjab		Overall Mean	
	CI	PI	CI *	PI	CI *	PI	CI *	PI
2HD-29	10.2	11.57	10.3	12.5	10.4	13.62	10.29 ^b^	12.58 ^d^
AARI-2011	10.9	16.3	10.3	11.41	10.5	12.32	10.44 ^b^	12.51 ^d^
Bakhtawar-92	10.3	12.66	10.3	11.89	10.6	13.49	10.36 ^b^	12.39 ^e^
Fakhre-Sarhad	10.3	13.22	10.9	14.8	10.7	14.29	10.73 ^a^	14.40 ^b^
Janbaz	10.2	11.47	10.3	12.9	10.4	12.74	10.32 ^b^	12.60 ^d^
NIFA-Barsat	10.6	14.42	10.2	11.5	10.3	12.44	10.28 ^b^	12.25 ^e^
Tatara	10.6	14.46	10.2	12	10.6	14.23	10.39 ^b^	12.95 ^c^
WL-711	10.7	16.1	10.6	13.5	11.7	19.89	10.88 ^a^	15.41 ^a^
Mean	10.5	13.77	10.4	12.56	10.6	14.13	10.5	13.14
LSD(0.01)							0.253	0.376

* Transformed data by adding 10. ^a–e^ Least significant difference lettering.

**Table 5:** Characteristics of different RAPD primers in *T. indica* isolates.

RAPD Primers	Primer 5′-3′	Total Bands	Monomorphic Bands	Polymorphic Bands	%Polymorphism	Unique Bands	Product Size Kb	PIC
OPA-3	AGTCAGCCAC	14	0	14	100	1(17)	0.25–2.0	0.95
OPA-9	GGGTAACGCC	9	0	9	100	0	0.28–1.5	0.95
OPA-13	CAGCACCCAC	11	0	11	100	0	0.25–1.5	0.87
OPA-18	AGGTGACCGT	11	0	11	100	1(61)	0.25–2.5	0.96
OPA-20	GTTGCGATCC	15	0	15	100	2(15, 30)	0.25–2.5	0.98
OPAA-1	AGACGGCTCC	11	0	11	100	0	0.25–1.2	0.97
OPAA-16	GGAACCCACA	13	0	13	100	0	0.25–2.0	0.94
RAPD-16	GTG AGG CGT C	15	0	15	100	0	0.25–2.5	0.8
Total		99	0	99	100	4	0.25–2.5	0.93

Values in parentheses of unique bands denotes number of code assigned to the isolates given in Table 1 and Table 2.

**Table 6:** Characteristics of different inter-simple sequence repeat (ISSR) primers in *T. indica* isolates.

Sr. No.	ISSR Primers	Primer 5′-3′	Total Bands	Monomorphic Bands	Polymorphic Bands	%polymorphism	Unique Bands	Product Size Kb	PIC
1	815	(CT)8G	13	0	13	100	0	0.3–3.0	0.970
2	812	(GA)(8)A	9	0	9	100	2(15, 58)	0.25–1.0	0.968
3	808	(AG)8C	14	0	14	100	0	0.2–1.2	0.762
4	ISSR-18	((CTC)(8))	10	0	10	100	0	0.2–1.2	0.973
5	864	(ATG)6	9	0	9	100	0	2.6–1.2	0.957
6	ISSR-1	(HVH(TG)(7))	6	0	6	100	1(41)	0.3–0.8	0.909
7	845	(CT)8RG	7	0	7	100	2(38, 44)	0.25–1.2	0.917
8	885	BHB(GA)(7)	7	0	7	100	0	0.26–1.2	0.947
9	847	(CA)8 A/G C	13	0	13	100	1(46)	0.25–1.3	0.955
10	817	(CA)8A	8	0	8	100	1(15)	0.26–2.0	0.895
11	876	(GATA)2(GACA)2	15	0	15	100	0	0.25–1.5	0.947
12	818	HBH(AG)7	8	0	8	100	0	0.25–1.0	0.965
13	890	VHV(GT)7	10	0	10	100	0	2.5–1.5	0.823
14	835	(AG)8YC	11	0	11	100	0	0.26–2.0	0.899
15	856	(CA)8 A/G C	13	0	13	100	1(1)	0.26–1.8	0.962
16	857	(AC)(8)YG	12	0	12	100	0	0.25–1.8	0.903
17	ISSR-17	(ATG)6)	12	0	12	100	0	0.28–2.0	0.698
18	810	(GA)8T	11	0	11	100	0	0.28–1.6	0.933
19	826	(AC)8C	12	0	12	100	0	0.28–1.5	0.821
20	856-2	(AC)8YA	15	0	15	100	1(17)	0.26–2.5	0.835
	Total		215	0	215	100	9	0.25–3.0	0.902

Values in parentheses of unique bands denote number of codes assigned to the isolates given in Table S3. V = A, C, G; B = G, C, T; H = A, C, T; D = A, G, T; Y = Pyrimidine (C, T); R = Purine (A, G).

**Table 7:** Summary of analysis of molecular variance (AMOVA) based on RAPD and ISSR of *T. indica* isolates.

Marker	Source of Variance	df	Sum of Squares	Mean Squares	Variance Components	Percentage of Variation	Fst	*p* Value
RAPD	Among the population	1	51.21	51.21	1.53	7.76	0.08	0.02
	Within the isolates	53	944.72	17.82	17.82	90.44		0.03
	Total	67	1248.69	18.64	19.71	100		
ISSR	Among the population	1	107.021	107.02	3.14	7.40	0.074	0.01
	Within the isolates	53	2049.5	38.66	38.66	91.0		0.02
	Total	67	2696.75	40.25	42.451	100		

Significant at *p* = 0.05.

**Table 8:** Summary statistic of mean genetic parameters based on RAPD and ISSR analysis of *T. indica* isolates.

Marker	Pop	N	MLG	eMLG	SE	H	G	Lambda	E.5	Hexp	Ia	rbarD	p.rD
RAPD	KPK	13	13	13	0.00	2.56	13	0.923	1	0.204	5.2	0.0452	0.001
	Punjab	55	55	13	2.12 × 10^−6^	4.01	55	0.982	1	0.2	5.32	0.0347	0.001
	Total	68	68	13	0.00	4.22	68	0.985	1	0.206	5.22	0.0321	0.001
ISSR	KPK	13	13	13	0.00	2.56	13	0.923	1	0.242	3.83	0.0149	0.001
	Punjab	55	55	13	2.12 × 10^−6^	4.01	55	0.982	1	0.209	5.59	0.0179	0.001
	Total	68	68	13	0.00	4.22	68	0.985	1	0.221	5.69	0.0175	0.001

Notes: Pop = A vector indicating the population factor, N = an integer vector indicating the number of individuals/isolates in the specified population; MLG = an integer vector indicating the number of multilocus genotypes found in the specified population; eMLG = the expected number of MLG at the lowest common sample size (set by the parameter minsamp); SE = the standard error for the rarefaction analysis; H = Shannon–Wiener Diversity index; G = Stoddard and Taylor’s Index; Lambda = Simpson’s index; E.5 = Evenness; Hexp = Nei’s gene diversity (expected heterozygosity); Ia = a numeric vector giving the value of the Index of Association for each population factor;, (see ia); rbarD = a numeric vector giving the value of the Standardized Index of Association for each population factor; (see ia); p.rD = *p*-value for r d.

**Table 9:** Comparison of RAPD and ISSR marker in evaluating genetic diversity of *T. indica* isolates.

Parameters	RAPD	ISSR
Number of assay(Primer) units	8	20
Number of total bands	99	215
Number of monomophic bands	0	0
Number of polymorphic bands	99	215
Percent polymorphism	100	100
Total number of unique bands	4	9
Size of PCR products	0.25–2.5 kb	0.25–3.0 kb
Fraction of polymorphic Marker(β)	1	1
Average PIC	0.92	0.90
Multiplex ratio (n)	1.547	0.537
Effective Multiplex ratio (E = βxn)	1.547	0.537
Marker Index (MI = Ex PIC)	1.43	0.482

## Data Availability

All the data are available as supplementary tables.

## Supplementary Materials

The following supporting information can be downloaded at: https://www.mdpi.com/article/10.3390/jof8030219/s1, Table S1, List of RAPID Primers used for the molecular characterization of *T. indica*; Table S2, List of ISSR Primers used for the molecular characterization of *T. indica*; Table S3, Disease reactions of eight wheat varieties to 49 isolates of *T. indica*; Table S4, Eigen value, explained variance and cumulative variance in the PCA using characters to classify 68 isolates by RAPD and ISSR; Table S5, Genetic structure of *Tilletia indica* isolates and estimates of gene flow within the genus.
