# Supplementary material for: Genetic Variability and Aggressiveness of Tilletia indica Isolates Causing Karnal Bunt in Wheat
Source: J Fungi (Basel). 2022 Feb 23;8(3):219. doi: 10.3390/jof8030219 (PMC8950583; doi:10.3390/jof8030219)
Supplement: Supplementary file 1 [file jof-08-00219-s001.zip › jof-1582175-supplementary.pdf]

Table S1 List of RAPID Primers used for the molecular characterization of *T. indica*

**Sr. No.**	**RAPD**	**Primer**	**Product size**	**Annealing temperatures** C^o	**Reference**
**1**	RAPID 16	GTG AGG CGT C	2.5-1.6 Kb	38	Gupta *et al*., 2015
**2**	OPA2	TGCCGAGCTG	0.3-1.1 Kb	36	
**3**	OPA3	AGTCAGCCAC	0.3-1.0 Kb	36	
**4**	OPA9	GGGTAACGCC	0.5-2.0 Kb	36	
**5**	OPA13	CAGCACCCAC	0.3-1.25 Kb	38	
**6**	OPA18	AGGTGACCGT	0.6-3.0 Kb	38	
**7**	OPA20	GTTGCGATCC	0.5-3.0 Kb	38	
**8**	OPAA1	AGACGGCTCC	0.4-2.0 Kb	36	
**9**	OPAA16	GGAACCCACA	0.3-3.0 Kb	38	
**10**	OPAA3	TTAGCGCCCC	0.5-3.5 Kb	36	Parveen *et al*., 2013
**11**	OPAA4	AGGACTGCTC	0.2-2.0 Kb	36	
**12**	OPAA7	CTACGCTCAC	0.1-2.5 Kb	42	
**13**	OPAA11	ACCCGACCTG	0.2-2.0 Kb	36	
**14**	OPAA13	GAGCGTCGCT	0.5-3.0 Kb	38	
**15**	OPAA15	ACGGAAGCCC	0.2-3.5 Kb	36	
**16**	OPAA17	GAGCCCGACT	0.2-2.0 Kb	36	
**17**	OPAA18	TGGTCCAGCC	0.4-2.0 Kb	36	
**18**	OPAA9	AGATGGGCAG	0.3-2.0 Kb	36	
**19**	OPAA10	TGGTCGGGTG	0.2-1.5 Kb	36	
**20**	OPAD10	AAGAGGCCAG	0.5-0.9 Kb	38	
**21**	OPAD12	AAGAGGGCGT	0.3-1.5 Kb	38	
**22**	OPAD5	ACCGCATGGG	0.2-2.0 Kb	38	
**23**	OPAD14	GAACGAGGGT	0.3-2.0 Kb	38	
**24**	OPC14	TGCGTGCTTG	0.4-3.0 Kb	38	
**25**	OPC8	TGGACCGGTG	0.7-1.5 Kb	36	
**26**	OPC15	GACGGATCAG	0.4-3.0 Kb	36	
**27**	OPB6	TGCTCTGCCC	0.3-3.0 Kb	36	

**28**	OPB8	GTCCACACGG	0.5-3.0 Kb	36
**29**	OPD2	GGACCCAACC	0.5-3.0 Kb	36
**30**	OPD7	TTGGCACGGG	0.4-3.0 Kb	36
**31**	OPD16	AGGGCGTAAG	0.4-3.0 Kb	36

Table S2 List of ISSR Primers used for the molecular characterization of *T. indica*

**Sr. No.**	**ISSR UBC**	**Primer**	**Product size KB**	**Annealing Temp.** C^{o}	**Reference**
1	826	(AC)8C 5'ACACACACACACACACC3'	0.3-1.2	54	
2	808	(AG)8C 5'AGAGAGAGAGAGAGAGC3'	0.3-0.7	54	
3	811	(GA)8C 5'GAGAGAGAGAGAGAGAC3'	0.3-1.2	54	
4	834	(AG)(8)T 5'AGAGAGAGAGAGAGAGT3'	0.3-0.75	50	
5	810	(GA)8T 5'GAGAGAGAGAGAGAGAT3'	0.15-1.1	50	
6	841	(GA)8YC 5'GAGAGAGAGAGAGAGAYC3'	0.15-1.1	58	
7	835	(AG)8YC 5'AGAGAGAGAGAGAGAGYC3'	0.3-0.7	54	
8	809	(AG)(8)G 5'AGAGAGAGAGAGAGAGG3'	0.3-1.0	54	
9	842	(GA)8YG 5'GAGAGAGAGAGAGAGAYG3'	0.2-2.0	58	
10	818	HBH(AG)7 5'HBHAGAGAGAGAGAGAG3'	0.35-1.5	50	
11	817	(CA)8A 5'CACACACACACACACAA3'	0.25-1.2	50	Parveen *et al.*, 2013
12	812	(GA)(8)A 5'GAGAGAGAGAGAGAGAA3'	0.5-2.5	50	
13	820	(GT)8C 5'GTGTGTGTGTGTGTGTC3'	0.7-3.5	54	
14	824	(TC)8G 5'TCTCTCTCTCTCTCTCG3'	0.3-2.5	54	
15	845	(CT)8RG 5'CTCTCTCTCTCTCTCTRG3'	0.3-1.1	52	
16	857	(AC)(8)YG 5'ACACACACACACACACYG3'	0.5-0.7	52	
17	864	(ATG)6 5'ATGATGATGATGATGATG3'	0.3-2.5	54	
18	873	(GACA)4 5'GAC AGA CAGACA GACA3'	0.3-0.7	54	
19	876	(GATA)2(GACA)2 5'GAT AGA TAGACA GACA3'	0.25-1.0	52	
20	880	(GGAGA)3 5'GGA GAGGAG AGGAGA3'	0.4-3.0	52	
21	881	(GGGTG)(3) 5'GGG TGGGGT GGGGTG3'	0.2-0.7	58	

22	890	VHV(GT)7<br>5'VHV GTGTGT GTGTGT GT3'	0.25-1.5	52	
23	840	(GA)(8)YT<br>5'GAGAGA GAGAGA GAGAYT3'	0.3-2.5	58	
24	815	(CT)8G<br>5'CTCTCT CTCTCT CTCTG3'	0.3-3.0	52	
25	847	(CA)8 A/G C<br>5'CAC ACA CAC ACA CAC AAC3'	0.3-1.0	54	
26	856	(CA)8 A/G C<br>5'CAC ACA CAC ACA CAC AGC3'	0.3-3.0	56	
27	856-2	(AC)8YA<br>5'ACA CAC ACA CAC ACA CYA3'		54	
28	844	(CT)8RC<br>5'CTC TCT CTC TCT CTC TRC3'	0.3-3.0	54	
29	885	BHB(GA)(7)<br>5'BHB GAG AGA GAG AGA GA3'	0.25-2.7	50	Gupta *et al.*, 2015
30	ISSR-18	((CTC)(8))<br>5'CTC CTCCTCCTCCTCCTCCTC CTC3'	500-1300	54	
31	ISSR-17	(ATG)6)<br>5'ATG ATGATGATGATGATG3'	500-1400	56	
32	ISSR-1	(HVH(TG)(7))<br>5'HVH TGT GTG TGT GTG TG3'	200-1400	50	

B= C,G,T; D=A, G,T; H=A,C,T;R=A, G;V=A,CG;Y=C,T

Table S3. Disease reactions of eight wheat varieties to 49 isolates of *T. indica*.

Isolates	WL-711	HD-29	Janbaz	Bakhtawar-92	Fakhre-Sarhad	Tatara	NIFA-Barsat	AARI-2011
PB-41	HR	R	HR	HR	R	HR	HR	HR
PB-42	R	R	HR	R	HR	HR	HR	HR
PB-43	HR	HR	HR	HR	HR	HR	HR	HR
PB-44	HR	R	HR	HR	R	R	HR	HR
PB-45	HR	R	HR	HR	HR	HR	HR	HR
PB-46	R	HR	HR	R	R	HR	R	HR
PB-47	R	R	R	R	R	HR	R	HR
PB-48	HR	HR	HR	HR	HR	HR	HR	R
PB-49	R	HR	HR	R	R	R	HR	HR
PB-50	HR	R	HR	HR	R	R	HR	HR
PB-51	R	R	R	HR	R	HR	HR	HR
PB-52	R	HR	R	HR	R	HR	HR	HR
PB-53	R	HR	HR	R	R	R	R	HR
PB-54	R	R	R	R	R	R	HR	HR

PB-55	MS	HR	HR	R	HR	R	R	R
PB-56	R	HR	R	R	HR	HR	HR	R
PB-59	R	HR	R	HR	R	HR	HR	HR
PB-60	HR	HR	HR	HR	HR	HR	HR	HR
PB-61	R	HR	HR	HR	HR	HR	HR	HR
PB-62	HR	HR	HR	HR	R	HR	HR	HR
PB-63	R	HR	R	HR	R	HR	R	HR
PB-64	HR	HR	HR	R	R	HR	HR	HR
PB-65	R	HR	HR	HR	HR	HR	HR	HR
PB-66	HR	HR	HR	R	HR	HR	HR	HR
PB-67	R	HR	HR	R	R	R	HR	HR
PB-68	HR	HR	HR	HR	HR	HR	HR	HR
PB-69	HR	HR	HR	R	R	HR	HR	HR
PB-70	R	R	HR	R	R	HR	R	HR
PB-71	HR	HR	R	HR	R	HR	HR	HR
PB-72	HR	HR	HR	HR	R	R	HR	R
PB-73	HR	HR	HR	HR	R	HR	HR	HR
PB-74	HR	R	R	HR	R	HR	HR	HR
PB-75	HR	HR	HR	HR	R	R	R	HR
PB-76	HR	HR	HR	HR	R	R	HR	R
PB-77	HR	R	R	HR	R	R	HR	HR
PB-78	R	HR	HR	HR	R	HR	HR	HR
PB-79	HR	HR	R	HR	HR	HR	HR	R
PB-80	HR	R	HR	HR	HR	HR	HR	HR
PB-82	HR	HR	HR	HR	HR	HR	R	HR
KPK-11	R	R	HR	R	HR	HR	HR	R
KPK-12	HR	HR	R	R	R	HR	HR	HR
KPK-13	HR	HR	HR	HR	HR	R	HR	R
KPK-14	HR	R	HR	HR	HR	HR	HR	HR
KPK-15	R	HR	R	HR	HR	R	R	HR
KPK-16	HR	HR	HR	R	R	HR	HR	R
KPK-17	R	HR	HR	HR	HR	R	R	HR
KPK-18	R	HR	HR	HR	HR	HR	HR	R
KPK-19	R	HR	R	R	HR	R	R	R
PB-25	MS	R	R	R	MS	R	R	R

Table S4 Eigen value, explained variance and cumulative variance in the PCA using characters to classify 68 isolates by RAPD and ISSR

Markers	PCA	Eigen value	Explained variance	Cumulative variance
RAPD	1	1.6656	14.89	14.89
	2	1.3043	9.128	24.013
	3	1.10131	6.508	30.521
ISSR	1	2.177	11.77	11.77
	2	1.57865	6.192	17.966
	3	1.45561	5.264	23.23

PCA: Principle component analysis

Table S5. Genetic structure of *Tilletia indica* isolates and estimates of gene flow within the genus

Marker	HT	Hs	GST	Gprime-st	D_het	Nm
RAPD	0.20783	0.19671	0.0534883	0.12641	0.02768	8.84784
ISSR	0.2308	0.21947	0.0491063	0.11994	0.02904	9.68199

: HT = Total diversity; HS = Diversity within population; D-het = Diversity between the population GST = coefficient of gene differentiation; G prime-st = Genetic differentiation according to Hedric (2005) Nm= gene flow based on GST
